# Structured narrative retell instruction for young children from low socioeconomic backgrounds: a preliminary study of feasibility

**DOI:** 10.3389/fpsyg.2014.00391

**Published:** 2014-05-08

**Authors:** Suzanne M. Adlof, Angela N. McLeod, Brianne Leftwich

**Affiliations:** Communication Sciences and Disorders, University of South CarolinaColumbia, SC, USA

**Keywords:** preschool, literacy, narrative language, listening comprehension, book reading, macrostructure, microstructure, feasibility

## Abstract

Successful acquisition of literacy depends on adequate development of decoding skills as well as broader, meaning-related knowledge and skills for text comprehension. Children from low socioeconomic status (SES) backgrounds are often challenged in both domains, relative to peers who are not economically disadvantaged. The efficacy of code-focused instructional programs for at-risk preliterate children is well supported, but less evidence is available regarding interventions to improve broader language and comprehension skills. This preliminary study tested the feasibility of a new intervention, “structured narrative retell instruction” (SNRI), and explored its potential to enhance meaning-related knowledge and skills, including vocabulary, listening comprehension, and narrative skills, in pre-literate, low SES children. SNRI used authentic children's books to model comprehension processes, explicitly teach story grammar, and implicitly target microstructural aspects of narratives. Participants included 9 children with a mean age of 60 months, who were randomly assigned to SNRI or to code-focused literacy instruction (CFLI). Each group received 12, 40-min instructional sessions over 6 weeks. Pre- and post-tests were administered to assess vocabulary, listening comprehension, narrative macrostructure and narrative microstructure, as well as alphabet knowledge, phonological awareness, and concepts of print. The feasibility of SNRI was demonstrated by completion of the designed study, moderately high treatment fidelity, and qualitative feedback from interventionists. The SNRI group also made significant gains on 4 of the 7 meaning-related measures (*p* < 0.10). In comparison, the CFLI group made significant gains on 2 of 7 meaning-related measures. We conclude that SNRI is feasible and shows potential for improving language skills related to comprehension and that further research investigating its efficacy is warranted.

## Introduction

Children from low socioeconomic status (SES) backgrounds have an elevated risk for language and reading difficulties and related academic consequences, as compared with peers from middle and upper class backgrounds (Brooks-Gunn and Duncan, 1997; McLoyd, 1998; Denton and West, 2002). Hair et al. (2006) reported that approximately one-fourth of all kindergarteners enter school with language development that is behind that of their peers, but children of poverty tend to carry this “risk” profile with higher frequency than their peers from mainstream environments. Moreover, these achievement gaps are observable across school grades. For example, Fiester (2010) reported that 83% of fourth-grade students from low-income families performed below proficiency standards on the National Assessment of Educational Progress (NAEP) reading test, as compared with 55% of students from moderate to high-income families.

Students who are from economically disadvantaged backgrounds may be especially challenged in their abilities to maintain levels of performance commensurate with peers from economically secure households as they progress through school (Chall and Jacobs, 2003; Pianta et al., 2008). Some evidence suggests that early-emerging disparities in educational attainment related to socio-economic factors may become more substantial over time due to a phenomenon referred to as the Matthew effect (Bast and Reitsma, 1998; Morgan et al., 2008; McNamara et al., 2011; Morgan et al., 2011; but see Baumert et al., 2012). The Matthew effect is demonstrated when children with weak initial performance are less able to benefit from learning opportunities than their higher-skilled peers (Burstall, 1978; Stanovich, 1986). Other research, while not specifically testing for the Matthew effect, emphasizes the stability of early literacy delays (Juel, 1988; Cunningham and Stanovich, 1997; Cabell et al., 2013). For example, in a recent longitudinal study of preschoolers from low SES homes, Cabell et al. (2013) found that emergent literacy profiles based on performance in the fall of the preschool year were highly stable, especially for highest and lowest achievers. Of the children in the lowest achievement group in the fall, only 21% moved into the average range by spring. Interestingly, those children who did make an upward shift in ranking had higher initial levels of oral language abilities. Taken together, these findings underscore the need for evidence-based interventions to close the gap for disadvantaged students early on, to develop a strong language and literacy foundation that yields success in school and life.

Successful acquisition of literacy depends on adequate development of decoding skills as well as broader language skills for text comprehension (Storch and Whitehurst, 2002). As a group, children from low SES backgrounds enter school with lower performance in both domains, including delays in alphabetic knowledge and phonological awareness, which support the acquisition of decoding abilities, and delays in meaning-related knowledge and skills, e.g., vocabulary, syntax, higher-level language skills, and world knowledge, which contribute to the development of reading comprehension (Hart and Risley, 1995; Lonigan et al., 1998; Hirsch, 2003; Durham et al., 2007; Huttenlocher et al., 2010). Research over the last three decades has provided strong evidence documenting the efficacy of interventions that explicitly and systematically target code skills in preschool-aged children of low SES backgrounds. Comprehensive reviews of research have concluded that explicit instruction in alphabet knowledge, phonological awareness, and letter-sound relationships leads to significant improvements in children's reading and spelling abilities, including children from low SES homes and other at-risk groups (National Reading Panel, 2000; National Early Literacy Panel, 2009; Piasta and Wagner, 2010). In contrast, there is less evidence for interventions that close performance gaps in the broader language skills that support the development of reading comprehension abilities, although a body of positive evidence is beginning to emerge (cf. National Early Literacy Panel, 2009; Fricke et al., 2013).

Shared book reading interventions are probably the most commonly studied oral language interventions for young pre-readers, as can be observed in recent meta-analyses by the National Early Literacy Panel (2009) and Swanson et al. (2011). The largest group of studies included in these syntheses has examined a method of shared storybook reading, termed “dialogic reading” in which parents or teachers try to engage a child in a conversation about the storybook during reading by asking open ended wh-questions, repeating the child's correct responses, and following the child's lead with the goal of having the child gradually take the lead in the book discussion (e.g., Lonigan and Whitehurst, 1998). Other shared book reading interventions have included a focus on teaching new vocabulary from storybooks (e.g., Beck and McKeown, 2007), using limited questioning to foster comprehension skills (e.g., Morrow, 1988) or computer-administered read aloud sessions (e.g., Verhallen et al., 2006). From these studies, it is clear that shared book reading activities help to improve children's vocabulary knowledge. However, relatively few studies examined effects for other aspects of oral language, such as syntax, narrative macrostructure and microstructure, and general listening comprehension (but see Reese et al., 2010). The two aforementioned research syntheses provided effect sizes for vocabulary compared to other oral language measures (e.g., norm referenced measures of receptive and expressive language, language sample measures, qualitative and quantitative measures of narrative retells). Although significantly positive effect sizes were obtained for oral language across studies [*ES* = 0.30 and *ES* = 0.35 in National Early Literacy Panel (2009) and Swanson et al. (2011), respectively], these effect sizes were smaller than those obtained for vocabulary (*ES* = 1.02 and *ES* = 0.60). Clearly, more research is needed to examine effects on multiple components of language, and identify interventions that have effects large enough to help close gaps for at-risk children.

The purpose of this study was to examine the feasibility of a new intervention program focused on retelling narratives from authentic children's books and to determine if it showed promise of effectiveness for improving meaning-related knowledge and skills in preliterate children from low SES backgrounds. We called this program “Structured Narrative Retell Instruction” (SNRI), and our rationale for developing it was based on previous research on shared book reading, as well as other research focused on children's comprehension and production of narratives.

First, research indicates that children's understanding of narrative structure develops prior to their comprehension of print and correlates with their later reading comprehension abilities (Bishop and Edmundson, 1987; Fazio et al., 1996; Kendeou et al., 2005; Oakhill and Cain, 2007; Dooley and Matthews, 2009; Wellman et al., 2011). These narrative skills are often weakened in children with language and learning difficulties (Merritt and Liles, 1987; Scott and Windsor, 2000; Cain, 2003; Fey et al., 2004). For example, a recent study of at-risk first grade children reported that responders and non-responders to brief code-based interventions showed significantly different performance on the pre-intervention assessments of narrative language skills (Allen et al., 2012). Second, narrative interventions involving story grammar analysis have generally been shown to be effective in improving reading comprehension skills in older, school-aged children (Dimino et al., 1995; National Reading Panel, 2000; Shanahan et al., 2010). Third, although relatively few studies have included preschool-aged children, there is some evidence that narrative-based language interventions can be successful for children who exhibit language impairments and other developmental delays (Petersen et al., 2010; Spencer and Slocum, 2010). Petersen (2011) reviewed narrative intervention studies involving children with language impairments or learning disabilities of the nine studies included in the review, three included at least some preschoolers in their participant sample (Tyler and Sandoval, 1994; Hayward and Schneider, 2000; Davies et al., 2004). Across all of the studies reviewed, children with language impairments benefited from narrative interventions, with gains observed in grammar skills, vocabulary, and narrative structure. Additionally, focusing on narratives during oral language intervention provides a medium for clinicians to target both lower- and higher-level language skills simultaneously, using both direct and indirect methods. For example, while eliciting narrative retells from children, clinicians can use modeling, expansion, prompting and elicited imitation to promote children's understanding and use of more advanced syntactic structures (e.g., Swanson et al., 2005).

Most previous studies targeting narrative skills in pre-readers have used researcher-designed narratives and/or wordless picture books to teach and elicit narratives (e.g., Hayward and Schneider, 2000; Davies et al., 2004; Swanson et al., 2005; Peña et al., 2006; Petersen et al., 2008; Spencer and Slocum, 2010; Allen et al., 2012; Green and Klecan-Aker, 2012;). These procedures help researchers provide greater control over the story structure and narrative components, as well as the length and linguistic complexity of narrative materials. However, we developed SNRI to focus on narrative stories from authentic published children's storybooks. Several factors led to this decision. The first involved ecological validity: If the ultimate goal is to improve later reading comprehension outcomes, it seems fitting to expose children to authentic texts as much as possible. As discussed previously, there is also a sizeable research base showing the benefits of shared book reading activities for vocabulary development (National Early Literacy Panel, 2009; Swanson et al., 2011; see also Cunningham and Zibulsky, 2011). Further, published children's storybooks often contain engaging artwork that can help make book reading and book discussions more enjoyable for young children. Lastly, although our study did not address home literacy activities, if our intervention was found to be effective, it is possible that it could be adapted in the future for implementation by parents in the home. We hypothesized that authentic children's books would likely be easier to incorporate into home literacy activities than experimenter-developed stories.

In an evidence-based practice framework, large-scale, fully powered randomized controlled trials (RCTs) are considered the “gold standard” for making treatment decisions (Rosen et al., 2006). However, these studies can be extremely costly in terms of human resources, time, and participant effort, leading some to suggest that careful consideration should be given to their usage (Sibbald and Roland, 1998; Stolberg et al., 2004). Consequently, it is prudent to establish the feasibility of an intervention during its development period, before proceeding with larger investigations. In the early phases of clinical outcomes research—including the process during which intervention is being developed—preliminary, small-scale investigations that are often exploratory in nature are important for helping researchers to determine if large-scale studies are viable pursuit (Robey, 2004; Fey and Finestack, 2009). From such investigations, researchers are able to assess whether or not the developed intervention can be implemented with fidelity within the target population, as well as whether the ideas and outcomes are able to be modified and sustainable (Bowen et al., 2009). Thus, the ultimate goal of our study was to determine whether a RCT investigating SNRI was warranted, or whether further development of the intervention was needed.

In sum, the goal of the current study was to determine whether the previously untested SNRI was feasible and whether it showed promise of being effective for improving meaning-related skills in at-risk, preliterate children. Our research questions included: (1) Is SNRI viable in its current form, or are modifications necessary prior to pursuing a larger-scale study? (2) Do children receiving SNRI show improvement in their vocabulary knowledge, narrative understanding, or narrative production skills? To address these research questions, we implemented SNRI within a childcare center that served low-income families. Because these participants could already be considered to be at risk for language, reading, and future academic difficulties due to economic disadvantage, we did not wish to withhold treatment from one group as a no-treatment control condition. Instead, we contrasted effects of SNRI with an intervention that focused on code skills, Code-Focused Literacy Instruction (CFLI). Which also used authentic children's storybooks but targeted letter knowledge, phonological awareness, and print knowledge. Previous research with preschoolers indicates that instruction in code-based skills does not generalize to comprehension skills and vice versa (e.g., Gamse et al., 2008; Bianco et al., 2010). These findings suggest that CFLI could serve as an appropriate, albeit conservative, control against which to compare the experimental SNRI. Our hypotheses were that (1) SRNI would be able to be implemented with fidelity, and (2) that children receiving SNRI instruction would be more likely to demonstrate gains in meaning-related knowledge and skills (i.e., on assessments of vocabulary, narrative understanding, and narrative production) than children receiving CFLI instruction.

## Methods

### Participants

Participants were recruited from a childcare center in Columbia, South Carolina, US that primarily served economically- disadvantaged families. Most of the families who patronized the childcare center received state assistance to pay for childcare while they worked or participated in job-related education or training. The center staff was asked to distribute study information and informed consent forms to parents of all enrolled children between 3 and 6 years of age (*n* = 25). Ten child participants aged 49–82 months (7 preschool, 2 kindergarten, 1 first grade) were initially recruited. All children were African American and spoke English as their first and only language. Prior to the instructional interventions, children were randomly assigned to receive either SNRI or CFLI treatment. One child from the SNRI group moved after the first week of intervention; thus, all study analyses focus on the five children in the CFLI group and four children in the SNRI group who completed all pre-test, intervention sessions, and post-test sessions. Each of these children had perfect attendance or made up any missed sessions prior to post-testing. Each child received free copies of each book used in the intervention, for a total of 10 books (two books were used in two intervention sessions each).

### Measures

Participants were presented a battery of assessments designed to characterize their individual language and cognitive profiles. Some tests were administered to provide a detailed profile of the children's speech, language and cognitive skills prior to the intervention, whereas others were used to measure change in targeted skills across the intervention period. All of the testing occurred in individual sessions in a quiet area of the center. The examiners were graduate students in speech-language pathology who had received training regarding the tests' administration and scoring procedures prior to meeting with the participants. A Ph.D. faculty member supervised each student examiner and was available on site to provide guidance and answer questions as needed. All sessions were audio recorded and video recorded for data analysis and reliability checking. Unless otherwise noted, all standardized assessments were double-scored by trained research staff to ensure reliability of scoring. All disagreements were resolved by discussion with the first author.

#### Pre-intervention descriptive measures

First, nonverbal cognitive abilities were measured with two subtests from the Leiter International Performance Scale-Revised (Leiter-R; Roid and Miller, 1997). The Figure Ground subtest measures a child's ability to identify an embedded figure within a complex picture. The Form Completion subtest measures a child's ability to recognize a whole picture from separate pictures of its parts. Next, basic receptive and expressive language abilities were assessed using the Basic Concepts and Parallel Sentence Production subtests of the Assessment of Language and Literacy (ALL; Lombardino et al., 2005). In the Basic Concepts subtest, participants are asked to point to pictures that represent concepts described by the examiner (e.g., size, location, comparison). The Parallel Sentence Production subtest evaluates a child's production of grammatical morphemes and syntactic structures. Following the procedures of the test manual, acceptable dialectical variations received full credit.

#### Pre-and post-test measures of intervention targets

***Receptive vocabulary***. The Peabody Picture Vocabulary Test, Fourth Edition (PPVT-4; Dunn and Dunn, 2007) was used to measure receptive vocabulary knowledge. For this test, the examiner presented a series of pictures, with four pictures per page, to the participant. The participant was required to point to the picture that was named or described by the examiner.

***Listening comprehension***. The preschool section of the ALL Listening Comprehension subtest (Lombardino et al., 2005) was presented. For this subtest, participants listened to three brief stories, each read twice by the examiner. After a story was read the first time, participants were asked to retell the story back to the examiner. Then the story was read a second time, and participants were asked to answer three to four comprehension questions about the story. Responses from the retells and comprehension questions were scored from audio recordings by trained research assistants according to the test manual, which awarded binary credit for inclusion of specific content elements from the story. The maximum possible score across the three stories was 21.

***Narrative macrostructure***. A finer-grained assessment of participants' narrative skills was obtained by transcribing and coding transcripts of participants' retells of the three preschool stories from the ALL Listening Comprehension subtest. To assess narrative macrostructure, each transcript was scored based on the following five components: characters, initiating event, character response, remaining events, and end of story. Each component received a score between 0 and 2, depending on the accuracy and amount of detail provided. For example, when all characters were referred to with nonspecific labels (e.g., “the boy” or “the girl”), one point was awarded for the character component, whereas when specific character names were used, two points were awarded. Scores for all components across all stories were summed, for a maximum possible score of 30. To ensure reliability of macrostructure scores, two trained research assistants coded macrostructure for each narrative. The correlation between total scores assigned by each rater was 0.99.

***Narrative microstructure***. To assess narrative microstructure, transcripts were coded and analyzed for length, lexical diversity, and grammatical complexity using SALT: Systematic Analysis of Language Transcripts software (Miller and Iglesias, 2012). Because the original stories were relatively short, the transcripts for the three stories were combined and analyzed as one unit. The total number of words across the three stories served as the outcome measure of length, and the number of different words served as the outcome measure of lexical diversity. Two measures were used to assess grammatical complexity, and both involved parsing the transcript into C-units. A C-unit is an independent clause (i.e., a “main clause”) with all its subordinate clauses. The first measure of grammatical complexity was the mean length of utterance (MLU), which was the average number of words per C-unit. The second measure of grammatical complexity was the clausal density, or the average number of clauses per C-unit. To achieve accurate and reliable microstructure measures, two trained research assistants separately entered the microstructure codes, compared them to each other, and settled discrepancies via discussion. Then, all transcripts were checked a third time by the first author.

***Alphabet knowledge***. The ALL Letter Knowledge subtest was used to measure alphabet knowledge. Test items required participants to point to, name, and write various lower- and upper-case alphabet letters. The maximum raw score for this subtest was 30.

***Phonological awareness***. The Rhyme Knowledge, and Sound Categorization subtests of the ALL were administered to assess participants' phonological awareness. The Rhyme Knowledge subtest includes four types of tasks, which required participants to determine whether two words rhymed, decide which word out of a set of words did not rhyme, produce a rhyming word when given a prompt, and complete a sentence with an appropriate rhyming word. The last type of task contains different items for preschool and kindergarten students than for first grade students. However, in order to compare participants' performance on the same set of items, we administered the preschool and kindergarten items to all participants. Thus, the maximum possible score for the Rhyme Knowledge subtest was 20. In the Sound Categorization tasks, the participants were asked to indicate which word out of a set of words did not begin with the same sound (phoneme) as the other words. The maximum possible score for this subset was 16.

***Print and book awareness***. The Book Handling subtest of the ALL was used as a measure of print awareness. In this subtest, participants handled a real children's book and were asked to identify book and print conventions, such as the cover of the book, the title, and the direction that the eyes move when reading. The maximum raw score for this subtest was 8.

### Procedures

This study took place over 8 weeks during the summer of 2012. Individual pre- and post-testing was completed during Weeks 1 and 8. In Weeks 2–7, participants completed twelve, 40-min sessions (two sessions per week) of CFLI or SNRI in small groups of two or three children. There were two small groups each for the CFLI and SNRI interventions. Each session was led by one graduate student clinician, assisted by an undergraduate student, and supervised by a Ph.D. faculty member. A total of four graduate students and two undergraduate assistants provided the interventions. To avoid possible effects of interventionists on the treatment outcomes, all interventionists were involved in the provision of both types of instruction. Each graduate student was responsible for leading one SNRI group and one CFLI group per week. Each of the undergraduate students assisted with two CFLI sessions and two SNRI sessions per week. All sessions were video and audio recorded for fidelity coding.

#### Book selection

All lessons for both CFLI and SNRI sessions were constructed with traditional children's picture books as the focus. Each session focused on a children's book selected as specifically appropriate for CFLI or SNRI interventions groups. SNRI books were true narratives, having characters, a setting, at least one problem, at least one attempt to solve the problem, and a resolution. (e.g., *Harry the Dirty Dog* by Zion, 1956). SNRI activities involved identification and discussion of these narrative components (i.e., story grammar components), explanations of advanced vocabulary, and practice retelling narratives. In contrast, CFLI books contained features that highlight sounds and print, such as use of rhymes, alliteration, onomatopoeia, and variable font sizes (e.g., *Chicka Chicka Boom Boom* by Martin and Archambault, 1989) and CFLI activities involved rhyming, wordplay, and letter-sound correspondence. A few storybooks fulfilled the requirements of each intervention type and were used in both groups and presented within the parameters of each condition (e.g., *Bear's Loose Tooth*, Wilson and Chapman, 2011). Regardless of the type of treatment condition being implemented, all sessions followed similar general routines. In each intervention, eight books were used for one session each, and two books were used for two sessions each. By the end of the intervention program, all participants had completed 12 lessons focused on 10 authentic children's books.

#### SNRI sessions

A sample lesson plan for the SNRI program is provided in Appendix A of the Supplemental Materials. At the beginning of each session, the clinicians introduced the book by reading the title and identifying the author and the illustrator and then leading a group discussion of the roles of the author (i.e., “to write the story”) and the illustrator (i.e., “create the pictures”). As sessions progressed, the clinicians requested that the participants describe the jobs of the author and illustrator with appropriate scaffolding as needed. The clinicians also encouraged story predictions based on the title and cover illustrations.

As the clinicians read the book aloud, they engaged participants in think-alouds and discussions of the story during the reading. Clinicians drew attention to story grammar components (e.g., characters, setting, problem, attempts to solve the problem, resolution) as they arose during the story, and visual supports unique to each story were used to help children remember them. For example, clinicians would draw pictures on a white board to represent the various narrative components or use puppets or small toy figures to represent characters in the story. Clinicians also paused during readings to explain pre-identified “Tier 2” vocabulary words (cf. Beck et al., 2002) and provide participants opportunities to discuss and act out word definitions.

After reading the story, the clinicians led the group in reviewing each of the components of the narrative by asking questions such as, “Who were the characters in this story?” Clinicians were instructed to make sure every child had an opportunity to respond to at least one question during the story. As answers were provided, clinicians provided modeling, prompting, recasts and expansions to encourage more complex language use. Next, each participant took a turn to retell the story, with clinicians using visual supports and verbal prompts, as needed, to scaffold the retelling. The clinicians used recasts and expansions of the participants' productions to encourage more complete and grammatically complex retells. A sample transcript of a child's scaffolded retell is provided in Appendix B of the Supplemental Materials.

Each session ended with a brief art activity related to the story, and participants were encouraged to sign their art with their name. Clinicians provided instructions and support as needed to complete the art activity, and they were encouraged to facilitate further discussion of the story when possible. Participants received their own copy of the book at the end of each session, and they and were encouraged to re-read the book at home with their family.

#### CFLI sessions

A sample lesson plan for the CFLI group is provided in Appendix C of the Supplemental Materials. CFLI sessions began with the clinicians leading the “alphabet song” while pointing at each letter on a visually appealing alphabet board. Each day, two or three “letters of the day” and “sounds of the day” (which corresponded to the letters) were then introduced. The clinicians named the alphabet letter, modeled how to write it on a white marker board, and modeled the pronunciation of the sound(s) it represented. Each participant was then provided with an opportunity to write the letter, name it, and say its sound(s) with scaffolding as needed.

After introducing target letters and sounds, the clinicians introduced the storybook with the similar procedures to the SNRI introduction. Discussions of the author and illustrator and their roles was the same. Instead of making predictions about the story, participants' attention was drawn to the letters and words of the title. Participants were directed to look for the “letter of the day” and listen for the “sound of the day” throughout the book reading.

As the clinicians read the story aloud, they drew the participants' attention to print concepts and portions of the book that featured rhymes or other wordplay. Participants were encouraged to be “rhyme detectives” and “sound detectives” and raise their hands when they heard rhyming words or words that started with the sound of the day. Clinicians were instructed to make sure every child was called on to answer a question related to each target in each session.

After the story, participants completed activities and games to increase phonological awareness and phonics knowledge. Activities included sorting pictures of rhyming words, categorizing words according to their initial or final sounds, and blending and segmenting words with manipulatives (e.g., tokens, foam alphabet letters, etc.). Activities were selected and modified, and clinicians provided scaffolding as needed, according to individual participants' skill progression throughout the intervention. A brief art activity related to the story closed each session, and participants were encouraged to sign their art with their name. Participants took home the storybook at the end of each session and were encouraged to share the book at home with their family.

#### Intervention fidelity

Prior to the initiation of the study, the clinicians and assistants completed a one-day training that covered the goals of the intervention, the participant information, and specific procedural expectations. During the training, clinicians practiced implementing instructional strategies for each intervention, and the faculty supervisors provided instruction and feedback for implementing each of the different interventions. Emphasis was placed on the importance of not introducing skills intended to be targeted in one type of instructional group into the other group.

To measure the degree to which the interventions were implemented as intended, a fidelity assessment checklist was developed for each intervention. The checklists included ratings for the presence and frequency of occurrence of each component of the intervention for each child in the group (see Appendix D and E in supplementary materials). All intervention sessions were videotaped, and a trained research assistant who was not involved in any other aspect of the project completed fidelity checks from the videos for eight sessions for each group (33% of all sessions). Fidelity was moderately high for each intervention [CFLI = 0.85 (0.08); SNRI = 0.78 (0.12)]. A primary reason for loss of fidelity was time spent managing problem behaviors within the sessions, which necessarily detracted from time spent on intervention goals. Although the mean fidelity rating for CFLI was slightly higher than for SNRI, the fidelity difference between conditions was not statistically significant (*p* > 0.2).

### Data analysis

Because the small sample size in this study makes it difficult to determine whether the obtained data meet the necessary assumptions of parametric analyses, nonparametric tests were used. First, a series of Mann–Whitney *U*-tests were run to compare language, literacy, and cognitive abilities of the two treatment groups prior to the intervention. Analyses of the descriptive measures (i.e., Form Completion and Figure Ground subtests of Leiter and Basic Concepts and Parallel Sentence Production subtests of the ALL) focused on standardized test scores. However, raw scores were used as the unit of analysis for the target outcome measures, given that several measures were not norm-referenced and that norms for most of the ALL subtests were not available for all participants.

Next, gains between pre- and post-test were calculated for each group separately, using Wilcoxon Signed Rank tests. Given our small sample size and the goal of determining whether the experimental intervention should be pursued with a future larger-scale study, we were more concerned about falsely retaining the null hypothesis than falsely rejecting it (cf. Robey, 2004). Therefore, we set alpha for two-tailed significance tests at 0.10, and no adjustments were made for repeated measures. Effect sizes for group comparisons and treatment effects were calculated using Cohen's *r* using the Z statistic from the nonparametric test and the formula *r* = *Z*$\sqrt{\text{N}}$ (Fritz et al., 2012).

## Results

### Descriptive measures

Table 1 provides descriptive statistics for chronological age and the background assessments of language and nonverbal cognitive ability for each group prior to the intervention. Mann–Whitney *U*-tests revealed no significant differences between groups on any of these measures. Based on effect sizes, the groups appeared to be well matched for chronological age and basic oral language, but somewhat less so for nonverbal cognitive ability.

**Table 1:** **Background assessments of age, oral language, and nonverbal cognitive abilities**.

	**SNRI median (Range)**	**CFLI median (Range)**	**Sig. test *N* = 9**	**Effect size *(r)***
Chronological age (months)	58.5 (49–82)	56.0 (50–71)	*U* = 9 *p* = 0.81	0.08
ALL basic concepts (SS)	8.0 (4–10)	9.0 (4–11)	*U* = 8 *p* = 0.62	0.17
ALL parallel sentences (SS)	9.0 (7–10)	9.0 (6–10)	*U* = 9 *p* = 0.80	0.09
Leiter figure ground (SS)	11.5 (6–13)	9.0 (7–12)	*U* = 6.5 *p* = 0.38	0.29
Leiter form completion (SS)	10.5 (9–15)	10.0 (7–12)	*U* = 7 *p* = 0.46	0.25

### Treatment effects for vocabulary, listening comprehension, and narrative skills

Descriptive statistics for each group are provided in Table 2 for the measures of vocabulary, listening comprehension, narrative macrostructure, and narrative microstructure. There were no significant group differences in pre-test scores for any measures, and effect sizes ranged from small to medium. Specifically, the between-group effect sizes for PPVT-4 (*U* = 9, *p* = 0.80, *r* = 0.08), ALL Listening Comprehension (*U* = 8, *p* = 0.62, *r* = 0.08), macrostructure score (*U* = 8, *p* = 0.62, *r* = 0.16), total number of words (*U* = 9, *p* = 0.80, *r* = 0.08), and number of different words (*U* = 10, *p* = 1.00, *r* = 0) ranged from nil to small, indicating that after the randomization of participants to groups and the attrition of one participant from the initial SNRI group, there were only small differences between groups on these measures at pretest. However, the between-group effect sizes for the grammatical complexity measures, MLU (*U* = 5, *p* = 0.22, *r* = 0.41) and clausal density (*U* = 5, *p* = 0.22, *r* = 0.41) were moderate, suggesting that the groups were less well matched on these measures at pre-test.

**Table 2:** **Pre- and post-test assessments of vocabulary, listening comprehension, narrative macrostructure, and narrative microstructure**.

	**SNRI group**				**CFLI group**			
	**Pre-test median (Range)**	**Median gain (Range)**	**Post-pre sig. test *n* = 4**	**Effect size (*r*)**	**Pre-test median (Range)**	**Median gain (Range)**	**Post-pre sig. test *n* = 5**	**Effect size (*r*)**
PPVT-4	64.5 (54–99)	7.5 (4–14)	*Z* = 1.83 *p* = 0.07	0.92	65.0 (42–100)	3.0 (–6 to 19)	*Z* = 1.10 *p* = 0.27	0.49
ALL listening comprehension	11.5 (4–16)	0.5 (–1 to 3)	*Z* = 0.82 *p* = 0.41	0.41	9.0 (1–16)	3.0 (–1 to 7)	*Z* = 1.63 *p* = 0.10	0.73
Narrative macrostructure	10.5 (6–20)	9.5 (0–13)	*Z* = 1.60 *p* = 0.11	0.80	13.0 (2–19)	6.0 (3–10)	*Z* = 2.03 *p* = 0.04	0.91
Total number of words	91.5 (63–132)	43.5 (24–58)	*Z* = 1.83 *p* = 0.07	0.92	112.0 (30–118)	28.0 (–6 to 47)	*Z* = 1.21 *p* = 0.23	0.54
Number of different words	57.5 (41–73)	16.0 (6–28)	*Z* = 1.83 *p* = 0.07	0.92	66.0 (22–75)	13.0 (–6 to 26)	*Z* = 1.22 *p* = 0.23	0.54
Mean length of utterance	7.42 (5.12–8.00)	0.18 (0.17–1.05)	*Z* = 1.84 *p* = 0.07	0.92	5.89 (4.29–7.00)	1.68 (–2.52 to 2.24)	*Z* = 0.67 *p* = 0.50	0.30
Clausal density	1.56 (1.13–1.71)	0.01 (–0.15 to 0.27)	*Z* = 0.37 *p* = 0.72	0.18	1.33 (1.20–1.38)	0.26 (0.13–0.43)	*Z* = 2.02 *p* = 0.04	0.90

Also shown in Table 2 are the significance tests and effect sizes for pre- to post-test comparisons for each group on each of these measures. The SNRI group showed large significant pre- to post-test gains on four of the seven measures, PPVT-4, number of total words, number of different words, and MLU (*r* = 0.92, *p* = 0.07 for all four measures); a large gain was also observed for the macrostructure measure, which was just above the criterion for significance (*r* = 0.80, *p* = 0.11). Non-significant gains were observed for the SNRI group for the ALL Listening Comprehension subtest and for clausal density, with medium (*r* = 0.41, *p* = 0.41) and small (*r* = 0.18, *p* = 0.72) effect sizes, respectively.

In comparison, the CFLI group showed significant improvement between pre- and post-test on two of the seven measures, narrative macrostructure and clausal density, with large effect sizes for both measures (*r* = 0.90 and 0.91, respectively, both *p* = 0.04), and a large and marginally significant gain for the ALL Listening Comprehension subtest (*r* = 0.73, *p* = 0.10). The CFLI group's effect sizes for the remaining four measures which showed non-significant gains (PPVT-4, total number of words, number of different words, and MLU) were in the moderate range (*r* = 0.30–0.54; *p* = 0.23–0.50) Overall, while both groups evidenced some gains, the SNRI group showed more statistically significant gains and more large effect sizes than the CFLI group. However, such results must be interpreted with caution, given the small sample sizes in the current study.

### Treatment effects for alphabet knowledge, phonological awareness, and print and book awareness

Measures of code skills were also administered for comparison purposes. Each group's performance on pre-tests and post-tests of code skills is summarized in Table 3. At pretest, there were no significant differences between groups on any of the measures, and all of the effect sizes were small, indicating that the two groups were well matched initially: ALL Letter Knowledge (*U* = 7.5, *p* = 0.54, *r* = 0.21), ALL Rhyme Knowledge (*U* = 9.0, *p* = 0.80, *r* = 0.16), ALL Sound Categorization (*U* = 9.0, *p* = 0.80, *r* = 0.08), ALL Book Handling (*U* = 9.0, *p* = 0.80, *r* = 0.08).

**Table 3:** **Pre- and post-test assessments of alphabet knowledge, phonological awareness, and print concepts**.

	**SNRI group**				**CFLI group**			
	**Pre-test median (Range)**	**Median gain (Range)**	**Post-pre sig. test *n* = 4**	**Effect size (*r*)**	**Pre-test median (Range)**	**Median gain (Range)**	**Post-pre sig. test *n* = 5**	**Effect size (*r*)**
ALL letter knowledge	26.0 (1–30)	0.5 (–2 to 7)	*Z* =0.55 *p* = 0.58	0.28	22.0 (6–30)	0.0 (–2 to 5)	*Z* = 1.07 *p* = 0.29	0.48
ALL rhyme knowledge	4.5 (0–18)	2.5 (–3 to 5)	*Z* = 1.10 *p* = 0.27	0.55	3.0 (2–15)	1.0 (0–14)	*Z* = 1.60 *p* = 0.11	0.71
ALL sound categorization	4.0 (1–7)	0.5 (0–2)	*Z* = 1.34 *p* = 0.18	0.67	5.0 (0–7)	3.0 (–2 to 7)	*Z* = 1.21 *p* = 0.23	0.54
ALL book handling	3.0 (0–4)	2.0 (0–4)	*Z* = 1.60 *p* = 0.11	0.80	2.0 (2–5)	2.0 (0–6)	*Z* = 1.60 *p* = 0.11	0.71

Although the test scores for some children in each group increased between pre- and post-test, differences between scores at pre- vs. post-test were not statistically significant for any measure for either group. However, the CFLI group obtained a large and marginally significant effect size for the ALL Rhyme Knowledge subtest (*r* = 0.71, *p* = 0.11), and both groups obtained large and marginally significant gains for the ALL Book Handling subtests (*r* = 0.80 and 0.71 for SNRI and CFLI, respectively; both *p* = 0.11). Effect sizes for the ALL Letter Knowledge and the ALL Sound Categorization subtests were in the moderate range and non-significant for both groups (*r* = 0.48–0.67; *p* = 0.18–0.58).

## Discussion

Children from low-SES homes are known to be at risk for reading difficulties, including learning to decode words and learning to comprehend texts. Although there is a large body of research to guide instruction in decoding, there is less evidence to inform instruction in the skills that support reading comprehension, especially for very young children. Therefore, the purpose of this preliminary study was to determine if SNRI is feasible and if it shows the potential to improve meaning-related knowledge and skills in at-risk, preliterate children. Specifically, we aimed to investigate the influences of this previously untested intervention on children's vocabulary knowledge, narrative comprehension, and narrative production and to determine if outcomes were sufficiently positive to warrant further investigation. Prior to initiating a large-scale randomized control trial, we also aimed to determine whether SNRI was feasible as designed and implemented as well as if modifications were needed to maximize treatment effects.

The participants were randomly assigned to receive either the experimental SNRI or the comparison CFLI in 40-min small-group sessions twice weekly for 6 weeks. Both intervention programs featured shared reading of authentic children's storybooks, with high amounts of interaction, though the topics of the interactions differed. Analysis of pre- and post-test measures of vocabulary knowledge, listening comprehension, narrative macrostructure, and narrative microstructure were administered to measure changes in meaning-related skills for each intervention group. In addition, measures of alphabet knowledge, phonological awareness, and book handling were also administered to measure changes in code skills.

### Treatment effects on meaning related skills

The SNRI group showed significant improvement between pretest and posttest on four of the seven meaning-related measures. Moreover, the SNRI group evidenced very large effect sizes for gains on five of the seven measures, which represented all but one type of the meaning-related skills we assessed. Specifically, we observed effect sizes of *r* = 0.80 or greater for assessments of vocabulary (PPVT-4), narrative macrostructure, narrative length (total number of words), lexical diversity (number of different words), and grammatical complexity (MLU). We were somewhat surprised that the SNRI group did not show significant improvement in ALL Listening Comprehension scores, given that they showed substantial gains in narrative macrostructure scores between pre- and post-test. However, the items on the ALL Listening Comprehension test were scored dichotomously based on children's responses related to selected specific details from each story, whereas the macrostructure scoring rubric was more closely aligned with the instructional components of SNRI (i.e., elements of story grammar) and awarded partial credit scores. Thus, the macrostructure score may have been a more sensitive measure of response to SNRI instruction than the ALL Listening Comprehension score.

The CFLI group also showed significant improvement on two of the seven meaning-related measures, with effect sizes above *r* = 0.7 for three of the seven measures: ALL Listening Comprehension, narrative macrostructure and clausal density. Thus, while the SNRI group showed improvement on a larger number of assessments than the CFLI group, the CFLI group showed improvement on two of the measures that the SNRI group did not. From these results, we conclude that SNRI shows some potential for improving a range of meaning-related skills in young, preliterate children, but further study with a larger sample is needed to determine whether the observed gains are reliable and whether they are significantly larger than those that would be obtained from other interventions.

### Treatment effects on code skills

The purpose of this study was to test an intervention that would support the development of children's oral and written language comprehension skills. However, we also included assessments of code skills, for comparison purposes and to measure the progress of the CFLI group on the targets of their intervention. Neither group made significant improvements on any of the code skill measures, although the CFLI group's gain for the ALL Rhyme Knowledge subtest was marginally significant with a large effect size. With the exception of the Book Handling subtest of the ALL, the effect sizes for pre- to post-test gains were generally in the moderate range. Both groups evidenced large and marginally significant effect sizes for gains on Book Handling. This result is not surprising, given that both interventions included an introduction to the day's storybook that involved discussion of the title, author, and illustrator. Overall, the two groups evidenced a similar pattern of results for code skills, with a slight edge for rhyme knowledge evidenced by the CFLI group. It remains to be determined whether such results would be replicated with a fully powered study.

### Evaluation of instructional methods in SNRI

This study builds on previous shared book reading interventions through the addition of explicit instruction on story grammar and focus on developing children's narrative macrostructure and microstructure during narrative retellings. SNRI sessions, which lasted 40 min, were also more intensive than the most common shared book reading intervention, dialogic reading, which averaged 10 min (Lonigan and Whitehurst, 1998). Although the effects of SNRI appear positive, future research should conduct a cost-benefit analysis to determine whether the extra intensity and additional treatment targets result in substantially better outcomes.

Our use of authentic children's books was also a variation on the work of earlier narrative language interventions in which wordless picture books were used for narrative interventions (Hayward and Schneider, 2000; Swanson et al., 2005) or in which the researchers designed the narratives (Davies et al., 2004; Spencer and Slocum, 2010; Green and Klecan-Aker, 2012). The participants appeared to enjoy the instructional sessions and looked forward to receiving storybooks that they could take home. Moreover, the treatment effects on meaning-related skills were positive for SNRI suggesting that the possible variation introduced by authentic storybooks does not mitigate treatment effects.

All of the clinicians provided written feedback regarding their experiences with implementing SNRI. Several remarked that prior to the intervention, they expected that SNRI would be easy to implement because it involved naturally engaging children in book reading. However, they discovered that teaching the story grammar elements was less intuitive and required more practice than they initially projected. Whereas the targets of the CFLI instruction, such as recognizing and generating rhymes, were relatively concrete, some of the story grammar components, such as problem and attempts to solve it, were more abstract and more difficult to explain to young children. Some interventionists reported that their comfort level with implementing the intervention varied depending on the specific story that served as the focus for the lesson. Additionally, while the use of visual supports that were unique to each story initially seemed to offer the most flexibility, the clinicians suggested that future studies try using consistent visual supports across all sessions. Although such visual supports would likely be more abstract, their consistency might facilitate participants' generalization and retention of story grammar components.

The interventionists were occasionally required to manage behavior within some of the groups. Competing environmental noise and distractions within the childcare center (staff and/or children interrupting the treatment session) also interfered with instructional time. Although the frequency of these factors was similar across treatment groups, they were perceived as more disruptive to the SNRI sessions than the CFLI sessions, perhaps because the activities of the SNRI sessions were less concrete than those of the CFLI sessions. For example, during the portion of the session where CFLI participants played card games or completed worksheets to practice code skills, the SNRI participants were retelling narratives. Thus, the clinicians suggested that the addition of manipulatives and “hands-on” activities be considered for future SNRI sessions. While some aspects of the intervention could be improved, generally, all interventionists were quite complimentary of their experiences and expressed an interest in seeing the development and testing of SNRI continue.

### Limitations

The current study has several limitations. First, the sample size was very small, which limited our statistical power for testing treatment effects and prevented us from using standard analyses to test for interactions between groups and treatment gains. To increase our power for detecting treatment effects, we used a more liberal critical alpha level (*p* < 0.10), which makes our findings more vulnerable to Type I error. Second, we did not have an untreated control group. Consequently, we cannot rule out the possibility that the both group's gains may have occurred due to normal maturation or simple practice effects. A third limitation is that it was not possible to conduct a longer-term delayed post-test to determine whether intervention effects were maintained. Given these limitations, the outcomes of this study should be interpreted with caution. However, the positive results we have observed support the undertaking of more rigorous experimental testing of the effectiveness of SNRI. Future studies should consider including a delayed posttest to determine if treatment gains observed immediately following treatment are maintained over a longer period.

## Conclusions and future directions

We aimed to determine the feasibility and potential effectiveness of SNRI for improving the comprehension-related skills of young, preliterate children from low SES backgrounds. The results of this study indicate that SNRI is feasible and shows promise of effectiveness. It builds on the findings of previous studies of shared book reading, which have primarily focused on vocabulary gains, as well as previous studies of narrative interventions, which have primarily focused on older school-aged children. This work is an initial step toward establishing evidence to address the paucity of data that exists regarding comprehension-related skills and examining narrative interventions among preschool and kindergarten children who are at-risk of reading failure. We conclude that future investigations on a larger scale are warranted, perhaps including modifications to enhance participants' success.

### Conflict of interest statement

The authors declare that the research was conducted in the absence of any commercial or financial relationships that could be construed as a potential conflict of interest.
