# Supplementary material for: Structured narrative retell instruction for young children from low socioeconomic backgrounds: a preliminary study of feasibility
Source: Front Psychol. 2014 May 8;5:391. doi: 10.3389/fpsyg.2014.00391 (PMC4021132; doi:10.3389/fpsyg.2014.00391)
Supplement: Supplementary file 1 [file DataSheet1.PDF]

Appendix A. Sample SNRI Lesson Plan for *Bear's Loose Tooth* (Wilson & Chapman, 2011)

1. **Introduce the story:**
    a. *The title of the book is* <u>*Bear's Loose Tooth.*</u> *I see a bear on the cover, and his mouth is open. The other animals are all looking at his mouth. I bet he has a loose tooth! Have you ever had a loose tooth?*
    b. *Let's see what else the cover of this book shows us. Right here is the author's name. The author of the book is Karma Wilson.*
    c. *Her job is to write the story. She wrote all the words in this book.*
    d. *The illustrator of the book is Jane Chapman.*
    e. *The illustrator's job is to draw the pictures. I really like the illustrations in this book.*
    f. *In this story, Bear's tooth is loose. He's worried he won't be able to eat with a loose tooth.*
2. **Read the story** fluently and with engaging prosody.
    a. Use think-alouds to scaffold story understanding.
        i. *Do you think Bear can eat with a loose tooth?*
        ii. *How are they going to ever get that tooth out of Bear's mouth?*
    b. Give child friendly definitions/demonstrations for difficult vocabulary, e.g.
        i. nibbled = taking little bites
        ii. gulped = making a loud noise when you swallow
        iii. gobbled = eating fast and noisily
        iv. fret = worry
        v. hare = a kind of rabbit
        vi. wren = a kind of bird
        vii. budge = move
        viii. nudge = a little push
        ix. curious = someone who is curious wants to learn more about something
    c. Point out narrative components as they arise.
        i. *Our most important character is Bear. But there are other characters in the story too. There's Mouse, and Hare, and Wren, and Owl, and Badger. They are all characters.*
        ii. *Does anyone remember our special word for where and when a story takes place? It's the setting. Where is this story taking place? That's right they are in a cave in the forest!*
        iii. *Here's our problem! The problem is that Bear needs to get his loose tooth out. I wonder how the characters will try to solve the problem.*
        iv. *So all the characters are trying to pull Bear's tooth out for him. That's how they're trying to solve the problem. Too bad they can't get it out.*
        v. *Now Bear's problem is solved! He pushed his tooth out with his tongue! How do you think he feels?*
3. **Engage children in a discussion of the story.**
    a. How come the animals had so much trouble getting the tooth out?
    b. Why do you think bear is dancing and grinning?
    c. Has the tooth fairy ever visited you? What has she left you?

4. **Review the story in preparation for child retellings.** Use the picture cards from the story to review portions of the story. Ask children to put the picture cards in order. Model a retelling of the story.
    a. *The story began in a cave in the forest. Bear was eating lunch and he realized he had a loose tooth. He was worried that he wouldn't be able to eat. Mouse told him not to worry because a new tooth would grow in its place. All the animals tried to help Bear get his tooth out, but it wouldn't budge. Finally bear wiggled it with his tongue, and it popped out. That night, Bear slept with the tooth near his head. The tooth fairy visited and gave him some blueberries. The next morning, when Bear was eating the blueberries he found another loose tooth!*
5. **Ask children to play the role of book critic.**
6. **Complete the art activity**. Make a bear puppet out of a paper bag and glue his teeth inside. Children can use the puppet to act out parts of the story.

Appendix B. Transcript of a scaffolded retell by a child in the SNRI group

Story: *Bear's Loose Tooth* (Wilson & Chapman, 2011)

E: So where should we start? Let's start with the title.
C: The title of the book.
E: What is the title?
C: The Bear Loose Tooth, by a main character.
E: Bear's Loose Tooth, and the bear is the main character. Right? Bear's Loose Tooth.
E: So Bear is the main character, and the setting of our story is in the forest, right? So it takes place in the forest.
C: So he ate lunch.
E: Who ate lunch?
C: The bear and his friends.
E: Oh, Bear and his friends ate a yummy lunch.
C: And he ate a cookie.
E: Remember, he gulped, he gulped and he gobbled? Gobble gobble gobble.
C: And he saw something wiggle.
E: Ooh he felt something wiggle?
C: Yes.
E: Did he feel something wiggle in his mouth?
C: A teeth! Teeth!
E: His tooth? His tooth, his tooth was loose?
C: I wish my tooth come out.
E: You want your tooth to come out?
C: Mmhmm.
[Discussion of child wanting to lose his own tooth]
E: Let's remember what happened in our story. So the bear is in the forest with his friends and they ate lunch.
C: Yeah, yeah.
E: And then something happened to bear's tooth.
C: Yep.
E: What happened?
C: His tooth, he point in his mouth and his tooth was wiggling.
E: And his tooth was wiggling, and then he was worried that he couldn't eat with his loose tooth, wasn't he?
C: Yes.
E: He was, he was.
C: He was so mad.
E: And then all the animals came and what did they do?
C: They pull it out, and pull it out.
E: They did. First they looked at the tooth, and they said, "Oh, it's wiggling." And then—oh the mouse told him something. The mouse said, "Don't worry." The mouse said something was going to happen to his tooth.
C: "Don't worry, your tooth will grow back."
E: Mmhmm, "You'll get a new tooth." And then the bear, he still needed to get his tooth out. That was the problem, right? The tooth wouldn't come out. So his friends helped him, right?

C: So the bird help him pull his leg out and his xxx.
E: So the bird used his leg to try to help bear pull the tooth out?
C: Yep.
E: Okay. Did it come out?
C: Nope.
E: Nope, it didn’t come out when the bird pulled it.
E: Did anyone else help the bear pull his tooth?
C: The owl said, “His tooth stuck.”
E: The owl said that the bird was too small, but did the owl pull the tooth out?
C: Nope.
E: Nope, the owl didn’t pull the tooth out. What about these animals?
C: They didn’t pull it out.
E: They didn’t pull the tooth out either. Nobody could get the tooth out, could they?
E: His tooth is still stuck.
E: And then what happened? Bear did something. What did bear do?
E: Remember he nudged…
C: Nudge…
E: Yeah, and that was a good nudge. The bear nudged the tooth, what did he use?
E: He nudged the tooth with his…
C: Tongue.
E: Mmhmm. With his tongue, he nudged the tooth with his tongue. And then what?
C: It was wiggling.
E: It was wiggling and then he nudged it with his tongue, and then what happened to that tooth?
C: It felled down.
E: It did, it fell out. He got the tooth out. His tooth fell out.
E: And then how did he feel?
C: He feel happy.
E: He was so happy. He was so happy, he danced and he grinned, do you remember he smiled?
C: Yes.
E: Mmhmm.
C: And he take the nap and put his teeth on the plate.
E: Uh huh. So he took a nap, and put his tooth right next to him on the plate.
C: And a prince put the berries on the bear plate.
E: The fairy came and put blueberries on his plate and took his tooth.
C: xxx xxx took his tooth.
E: The fairy took his tooth. And then he woke up and what did he eat for breakfast?
C: Berries.
E: He ate the blueberries for breakfast. And then what happened? He gulped again remember? Gulp gulp gulp gulp gulp.
C: Gulp gulp gulp gulp gulp.
E: Gobble gobble gobble gobble gobble
C: Gobble gobble gobble gobble gobble.
E: And then what happened? Uh oh!
C: His ‘nother teeth came out.
E: Then he had another loose tooth.
C: They said “that was funny.”

E: Is that what they said at the end? Is the story over?
C: I think so.
E: You think so? So let's say, "The end."
C: The end.
END

Appendix C. Sample CFLI Lesson Plan for *Bear's Loose Tooth* (Wilson & Chapman, 2011)

1. **Alphabet Song:** Sing the alphabet song and point to letters on the chart as you sing.
2. **Letters and sounds of the day:** B and T; /b/ and /t/
    a. Review letters and sounds from last lesson (S and C; /s/ and /k/).
    b. Introduce new sounds: *Today we are going to talk about the letter 'B'. When we see the letter 'B', we know that it stands for the /b/ sound. So, we say B makes the /b/ sound. Can you say the /b/ sound? Say it with me /b/.* (Note that /b/ is a stop consonant; try not to over-emphasize a vowel sound when demonstrating the sound.) *B comes at the beginning of the word "bear." Say it with me "bear." The letter "B" looks like this* [draw it on the white board]. *Who would like to try to write a B on my white board? You do! Okay, come write a B on my board. While he's writing the B let's all say the /b/ sound together. Very good!* [repeat so that all children have a chance to participate, and then move on to the next letter/sound.]
3. **Introduce the story:**
    a. The title of the book is <u>Bear's Loose Tooth.</u> I see a bear on the cover, and his mouth is open. The other animals are all looking at his mouth. I bet he has a loose tooth! Have you ever had a loose tooth?
    b. *Let's see what else the cover of this book shows us. Right here is the author's name. The author of the book is Karma Wilson.*
    c. *Her job is to write the story. She wrote all the words in this book.*
    d. *The illustrator of the book is Jane Chapman.*
    e. *The illustrator's job is to draw the pictures. I really like the illustrations in this book.*
4. **Read the story** fluently and with engaging prosody. Before reading, tell the children you're looking for rhymes in the story. *This story has a lot of rhymes. It has a lot of words that sound like each other. Words that rhyme sound almost exactly the same at the end, but they're still different words at the beginning. Listen to my story really closely, and see if you can find some rhymes!*
    a. While reading, pause at least three times for each of the following:
        i. Concepts of print (e.g., the big font for the refrain "Bear's loose tooth" tells us to say it a little louder than the other words; the exclamation points tell us the author or character is excited)
        ii. Occurrences of letters of the day (e.g., Bear, tooth)
        iii. Occurrences of the sound of the day (e.g., Bear, tooth)
        iv. Rhyming words, e.g.,
            1. First page (munch, munch, crunch, lunch).
            2. "Wren perched on bear's **lip** and he got a good **grip**. . ."
            3. "That night in **bed**, right next to his **head**"
5. **Phonological awareness activities**: There are two phonological awareness activities for this book, a rhyme sort (e.g., sort the pictures into groups of words that rhyme), and an initial sound sort.
    a. *Look at all these pictures. I see lots of words that rhyme in these pictures. Let's see if we can sort these pictures into groups of words that rhyme.*
    b. You can adjust difficulty of the sort as needed.
        i. Decrease difficulty by limiting number of cards/choices.

ii. Increase difficulty/interest by using cards for a memory or go-fish game.

6. **Art activity**. Make a bear puppet out of a paper bag.
    a. While they are gluing on each of the bear's parts, ask children to identify or generate a rhyming word (e.g., eyes/pies, ear/cheer) or a word that starts with the same sound (mouth/mouse, teeth/top).
    b. Each child should autograph his/her art project; provide scaffolding as needed.

Appendix D. SNRI Fidelity Assessment

SNRI Fidelity Scoring | **Session:** ________________

Fidelity Coder: __________________________ | **Book:** __________________________________

Coding Date: ____________________________ | **Clinicians:** ______________________________

Directions: Circle 1 score (0, 1, 2 or 3) for each numbered item in table.

Book Reading **start time on video:**				
1. Introduces story title (1), describes cover (1), and makes a prediction or asks children what the story will be about (1)	0	1	2	3
2. Introduces author (1), author's job (1) and requests a response about author from student (1)	0	1	2	3
3. Introduces illustrator (1), illustrator's job (1), and requests a response about illustrator from student (1)	0	1	2	3
4. Every child was given a chance to respond to title, author, or illustrator.	0	1		
5. Uses "think alouds" to scaffold understanding of difficult story concepts (0=never, 1=sometimes, 2=frequently).	0	1	2	
6. Uses recasts and expansions to model sophisticated language use (0=never, 1=sometimes, 2=frequently).	0	1	2	
7. Gives child friendly definitions for difficult vocabulary words (1 pt each time).	0	1	2	3
8. Explicitly points out the characters of the story.	0	1		
9. Explicitly draws attention to the setting of the story.	0	1		
10. Explicitly draws attention to the problem(s) of the story, or what happens first, second/next, etc.	0	1		
11. Explicitly draws attention to the way the problem is solved, or how the story ends.	0	1		
Discussion after reading **start time on video:**				
12. Asks questions to review important story components and probe comprehension from students (1 pt per question/discussion statement).	0	1	2	3
13. Each child has an opportunity to respond/participate in discussion.	0	1		
14. Reviews characters (1) and uses word "character" (1)	0	1	2	
15. Reviews setting	0	1		
16. Reviews problem (or what happened first)	0	1		
17. Reviews attempt to solve (or what happened next)	0	1		
18. Reviews resolution (or how the story ended)	0	1		
19. Every child had a chance to answer at least one question in 14-18.	0	1		
20. Every child had a chance to be the "book critic."	0	1		
21. Clinician models retelling of the story	0	1		
Child Retellings **start time on video:**				
22. Each child has an opportunity to retell the story	0	1		
23. Clinician uses prompting, recasts, and expansions to help scaffold children's retellings. (0=never, 1=sometimes, 2=always).	0	1	2	

Art Activity **start time on video:**				
24. Clinicians continue discussing/re-enacting story during art activity	0	1		
**Session End time on video:**				
**Column Totals**	0			
**Raw Score (sum of all Columns)**				
**Fidelity Score** = Raw Score /38				

NOTES:

Appendix E. CFLI Fidelity Assessment

CFLI Fidelity Scoring **Session:** ________________

Fidelity Coder: __________________________ **Book:** _________________________________

Coding Date: ____________________________ **Clinicians:** _____________________________

Directions: Circle 1 score (0, 1, 2 or 3) for each numbered item in table.

Introductory Activities				
1. Sings alphabet song (1) and points to letters while singing (1)	0	1	2	
2. Reviews letters and sounds from previous sessions	0	1		
3. Each student has an opportunity to respond to letters/sounds from previous sessions	0	1		
4. Introduces first letter of the day (1) and sound of the day (1)	0	1	2	
5. Each student has an opportunity to write first letter of the day	0	1		
6. Introduces second letter of the day (1) and sound of the day (1)	0	1	2	
7. Each student has an opportunity to write second letter of the day	0	1		
Book Reading **start time on video:**				
8. Introduces story title (1), describes cover (1), and makes a prediction or asks children what the story will be about (1)	0	1	2	3
9. Introduces author (1), author's job (1) and requests a response about author from student (1)	0	1	2	3
10. Introduces illustrator (1), illustrator's job (1), and requests a response about illustrator from student (1)	0	1	2	3
11. Every child was given a chance to respond to title, author, or illustrator.	0	1		
12. Explicitly draws attention to concepts of print (1 pt each time)	0	1	2	3
13. Every child was given a chance to respond to concepts of print	0	1		
14. Explicitly draws attention to letters of the day (1 pt each time)	0	1	2	3
15. Every child was given a chance to respond to letters of the day	0	1		
16. Explicitly draws attention to sounds of the day (1 pt each time)	0	1	2	3
17. Every child was given a chance to respond to sounds of the day	0	1		
18. Explicitly draws attention to rhymes and/or same initial sounds (1 pt each time)	0	1	2	3
19. Every child was given a chance to respond to rhymes and/or same initial sounds	0	1		
Phonological Awareness Activities **start time on video:**				
20. Each child has 3 opportunities to respond during activity focused on rhyming (1 pt each opportunity given to **<u>all</u>** children in group)	0	1	2	3
21. Each child has 3 opportunities to respond during activity focused on same initial sounds (1 pt each opportunity given to **<u>all</u>** children in group)	0	1	2	3
22. **In sessions 5-12 only**, each child has 3 opportunities to respond to a higher-level phonological awareness activity (i.e., blending, segmenting, deletion, phonics; 1 pt each opportunity given to **<u>all</u>** children in group)	0	1	2	3
Art Activity **start time on video:**				
23. Explicitly draws attention to phonological awareness targets during art activity (1 pt each time)	0	1	2	3
24. Every child was given a chance to respond to rhymes and/or same initial sounds during art activity	0	1		

25. Every child signed name on art activity.	0	1		
**Session End time on video:**				
**Column Totals**	0			
**Raw Score (sum of all Columns)**				
**Sessions 1-4 Fidelity Score** = Raw Score /47				
**Sessions 5-12 Fidelity Score** = Raw Score /50				

NOTES:
